# Multifunctional ligand engineering for pure-blue halide perovskite nanocrystal LEDs

**DOI:** 10.1038/s41377-026-02288-4

**Published:** 2026-04-20

**Authors:** Mark T. Swihart

**Affiliations:** Department of Chemical and Biological Engineering and RENEW Institute, University at Buffalo (SUNY), Buffalo, NY USA

**Keywords:** Nanoparticles, Inorganic LEDs

## Abstract

More efficient and stable blue LEDs are essential for achieving the full potential of halide perovskite-based displays, but defect formation and ion migration limit both external quantum efficiency and lifetime of these devices. Now, a multifunctional fluorinated ligand is shown to mitigate both factors, dramatically enhancing brightness, efficiency and lifetime.

## Abstract (web summary)

Passivation with a bifunctional fluorinated ligand stabilizes mixed-halide perovskite nanocrystals, enabling high-color-purity deep-blue emission (460–470 nm) in LEDs with improved efficiency and lifetime vs. the state of the art.

## Graphical abstract

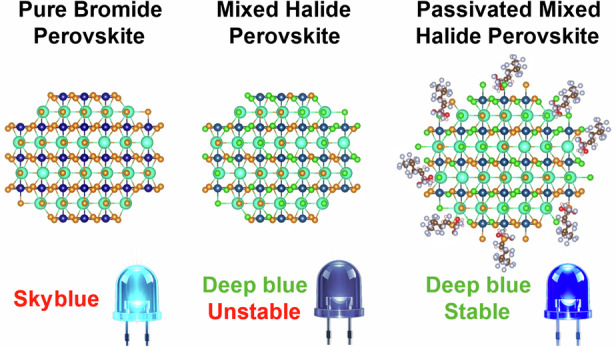

Perovskite LEDs (PeLEDs) with halide perovskite nanocrystals (PeNCs) as their emitting layers could be used to create displays with higher color purity and wider color gamut than current OLED and QD-LED displays^1^, as illustrated in Fig. 1a. This is a direct result of the narrower emission spectra (FWHM of 10–40 nm, Fig. 1b) of PeNCs compared to organic fluorophores or conventional II-VI or III-V quantum dots^1,2^. However, practical implementation of such displays has been limited by low external quantum efficiency (EQE) and instability of PeLEDs^2–4^. While red and green PeLEDs had already exceeded 20% EQE by 2020, and continue to improve, the EQEs of deep blue LEDs that are also needed to achieve a broad color gamut have remained much lower^5^. Moreover, these deep blue LEDs are notoriously unstable with device lifetimes measured in seconds to minutes^2,4^. Now, writing in Light: Science & Applications, Maimaitizi et al.^6^ show that passivating mixed-halide CsPb(Br/Cl)_3_ PeNCs with a multifunctional fluorinated linker can both passivate surface vacancies and stabilize halogen ions to suppress ion migration, significantly enhancing both EQE and device lifetime.

**Fig. 1:**
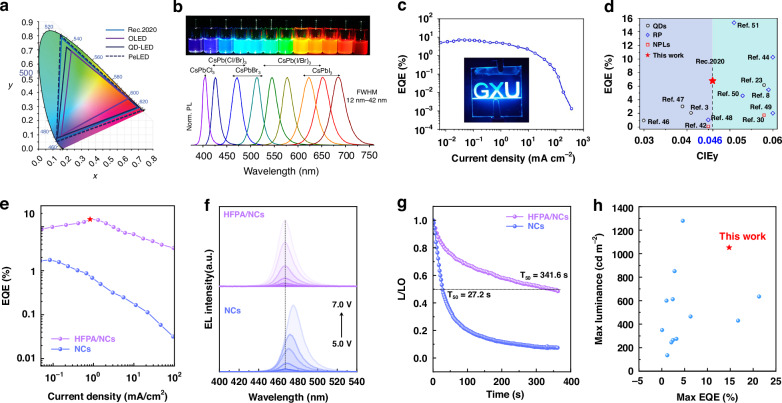
Background and advances in deep blue PeLEDs. **a** Illustration of the CIE color space comparing the area that can be covered by OLED, QD-LED, and PeLED technologies, along with the Rec.2020 standard^2^. **b** Illustration of emission colors achievable with cesium-lead-halide PeNCs of varied halide content^10^. **c** EQE vs. current density for the device reported by Song et al.^8^ and (**d**) benchmarking of that device vs. prior reports in terms of CIEy parameter and EQE (see ref. ^8^ for details). **e** EQE vs. current density for the devices reported by Maimaitizi et al.^6^, using PeNCs with (HFPA/NCs) and without (NCs) passivation with HFPA. **f** Dependence of electroluminescence spectrum on voltage, and (**g**) normalized electroluminescence intensity decay with time for these devices. **h** Benchmarking of the device using HFPA in terms of luminance and EQE vs. prior reports

As shown in Fig. 1b, CsPbBr_3_ PeNCs emit at wavelengths longer than the 460 to 470 nm range needed for the pure blue component of high color purity displays. CsPbCl_3_ PeNCs emit at wavelengths shorter than the desired range but suffer from instability due to the low formation energy of Cl^−^ vacancies^7^. This leads to two primary strategies for achieving the desired pure blue emission: (1) use of mixed-halide CsPb(Br/Cl)_3_ PeNCs, or (2) use of ultrasmall CsPbBr_3_ PeNCs in which quantum confinement effects widen the band gap and thus shift emission to shorter wavelengths^2,3,5^. The latter strategy is exemplified by the work of Song et al.^8^ who synthesized quantum-confined CsPbBr_3_ nanoplatelets (NPLs) with emission peaking at 461 nm, a narrow FWHM of only 13 nm, and impressive photoluminescence quantum yield (PLQY) of 96%. This allowed them to demonstrate a deep blue PeLED with the color purity needed for next-generation displays (CIE color coordinates of (0.135, 0.046, meeting the REC.2020 standard), but the EQE was still below 7% (Fig. 1c, d). The NPLs themselves exhibited impressive stability, and devices using CsPbBr_3_ are expected to be more stable than those using CsPb(Br/Cl)_3_ PeNCs, but device stability was not fully characterized. Huang et al.^9^ earlier reported quantum-confined CsPbBr_3_ NPLs and a device meeting the REC.2020 standard, but with lower EQE.

The strategy of using mixed halide CsPb(Br/Cl)_3_ PeNCs is the most straightforward route to achieving deep blue emission. Peak wavelength is readily tuned by varying the Br/Cl ratio rather than relying on quantum confinement. However, these mixed-halide PeNCs have been plagued by defects and instability^4^. Numerous doping and passivation strategies have been applied to stabilize them^2,3,5^. For example, Long et al.^7^ achieved impressive results using formamidinium doping of CsPb(Cl_0.5_Br_0.5_)_3_ PeNCs, including a maximum luminance of 1452 cd m^−2^, but with an EQE of 5% and peak emission wavelength of 474 nm, slightly outside the desired range. Thus, ligand engineering strategies to improve the performance and stability of CsPb(Br/Cl)_3_ PeNCs continue to be of great interest.

The key ligand engineering advance in the new report by Maimaitizi et al.^6^ is the use of a fluorinated multifunctional passivating agent, (1H,1H,2H,2H-heptadecafluorodec-1-yl)phosphonic acid (HFPA), that interacts with the CsPb(Br/Cl)_3_ PeNCs via multiple mechanisms. As in other examples of ligand engineering, HFPA displaces the oleic acid (OA) and oleylamine (OAm) ligands used in the PeNC synthesis. HFPA interacts with uncoordinated Pb^2+^ on the PeNC surface through its phosphonate groups, while simultaneously interacting with adjacent halide ions via hydrogen bond formation and stabilizing the halide octahedra via interactions with its fluorine atoms. Insights into these interactions are provided by DFT studies. Importantly, this ligand is both strongly bound and short enough to promote effective charge transfer to and from the PeNCs. Ultimately, this serves to stabilize surface halide ions, inhibiting ion migration and the formation of Cl^-^ vacancy defects. Compared to unmodified (OA/OAm capped) PeNCs in the same device structure, this ligand engineering strategy using HFPA enhances EQE, maximum luminance, and device half-life, by factors of 9, 10, and 13, respectively, as illustrated in Fig. 1e, f. This ligand engineering also stabilizes the emission wavelength, as illustrated in Fig. 1g, which shows the typical red-shift of emission with increasing drive voltage for the unmodified PeNCs and the absence of this shift for the HFPA modified PeNCs. While the final device has neither the highest EQE or highest luminance reported for deep blue PeLEDs, its combination of luminance and EQE represents a notable advance over the state-of-the-art, going beyond the pareto front of prior reports, as illustrated in Fig. 1h. The study is also notable for the thoroughness of its characterization of the PeNCs and their photoluminescence and electroluminescence properties, which provides clear insights into the origins of the differences between the unmodified and HFPA-modified PeNCs.

In summary, the report by Maimaitizi et al.^6^ is a notable advance in the development of CsPb(Br/Cl)_3_ PeNCs for deep blue PeLEDs and marks a step toward their practical implementation. That being said, many challenges remain. PeLED performance is determined by many other factors in addition to the PeNCs in the emissive layer. The overall device structures, while similar across different reports on deep blue PeLEDs, differ in detail, limiting the ability to make direct comparisons across studies. Among other factors, EQE depends on the efficiency and balance of charge injection into the emissive layer from the hole- and electron-transfer layers and the outcoupling of light from the device. Selecting the hole- and electron transport layer materials is increasingly challenging with increasing band gap of the emissive layer. Likewise, short device lifetimes are most often attributed to the emissive layer but may also result from degradation of other device layers and, particularly, the interfaces between them. Extending device lifetimes is perhaps the greatest remaining challenge; even with improvements enabled by ligand engineering, the device reported by Maimaitizi et al.^6^ has a half-life of less than six minutes, operating in an inert atmosphere glove box. This remains far from a practical device. While PeLEDs are becoming competitive with OLEDs with respect to EQE and luminance, and outperform OLEDs with respect to color purity, the half-lives of commercially relevant OLEDs are measured in thousands of hours. Further innovations in materials, device fabrication, and encapsulation will be needed to close this gap.
